# Carbonate-Metal Reactions in the Lower Mantle

**DOI:** 10.1021/acsearthspacechem.3c00101

**Published:** 2024-03-25

**Authors:** Anne H. Davis, Bethany A. Chidester, Eran Greenberg, Vitali B. Prakapenka, Andrew J. Campbell

**Affiliations:** † Department of the Geophysical Sciences, The University of Chicago, 5734 S. Ellis Avenue, Chicago, Illinois 60637, United States; ‡ Los Alamos National Laboratory, Los Alamos, New Mexico 87545, United States; § Center for Advanced Radiation Sources, The University of Chicago, 5734 S. Ellis Avenue, Chicago, Illinois 60637, United States

**Keywords:** high-pressure, diamond anvil cell, X-ray diffraction, SEM, FIB, carbonates, redox reactions

## Abstract

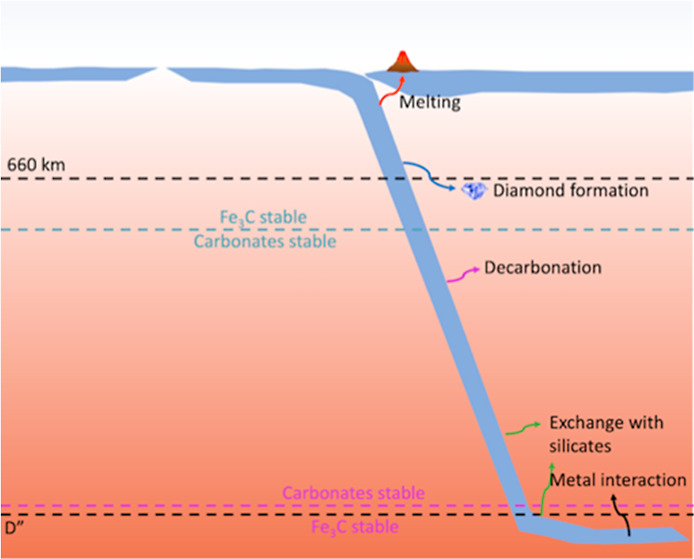

Carbonates are important carbon-bearing phases in the mantle. While their role in upper mantle petrologic processes has been well studied, their effect on phase relations, melting, and transport properties in the lower mantle is less understood. The stability of carbonates in the mantle depends on a host of factors, including pressure, temperature, oxygen fugacity, and reactions with surrounding mantle phases. To understand the stability of carbonates in the presence of metal in the lower mantle, carbonate-metal reaction experiments on the Fe–Si–Ca–Mg–C–O system were conducted up to 124 GPa and 3200 K. We find that carbonates react with iron alloys to form silicates, iron carbides, and oxides. However, the temperature at which these reactions occur increases with pressure, indicating that along a geotherm in the lowermost mantle carbonates are the stable carbon-bearing phase. Carbon is found to be less siderophilic at high-pressure compared to silicon.

## Introduction

The storage and cycling of carbon are intimately connected to Earth’s formation and development throughout geologic time. Carbonates are components of subducting slabs, making them important carriers of carbon into the mantle and important players in the deep Earth carbon cycle.^1,2^ In the upper mantle, carbonates are known to undergo a host of processes, including pressure-induced phase transitions,^3−5^ melting to form carbonatite melts,^6−8^ decarbonation reactions with free silica,^9−11^ and redox reactions with silicates, metals, and oxides to form diamond and/or carbide.^12−14^ Studies on carbonates and their stability in upper mantle phase assemblages have been plentiful.^15−19^ However, similar petrologic studies on carbonates in the lower mantle are lacking, with a few notable exceptions.^20−22^

The dearth of petrologic lower mantle carbonate studies stems partly from differing views on the amount of carbon contained in the lower mantle and the stability of carbonates under lower mantle conditions. Out of an estimated total of 62 megatons of carbonate subducted per year,^23^ estimates on the amount of carbon contained in carbonates that is transported into the lower mantle vary from 0.0001 to 52 megatons yearly,^1,24^ and estimates of primordial carbon contained in the mantle vary from 30 to 1000 ppm.^1^ Additionally, carbonate stability has been shown to depend on pressure,^25,26^ temperature,^27,28^ and oxygen fugacity.^14,29^ Under reducing conditions, carbonates have been shown to reduce to diamond and/or carbide phases.^13,20,22^ The instability of carbonates under reducing conditions has led to the redox-freezing hypothesis,^14,30^ which suggests that the mantle becomes increasingly metal-saturated and reducing with depth, forcing carbon to transition from an oxidized to a reduced form.

Alternatively, it can be argued that because the mantle is chemically heterogeneous,^31^ carbonate stability may depend on redox conditions set by local chemistry. Carbonate inclusions in deep Earth diamonds^32,33^ and constraints on the kinetics of carbonate-metal redox reactions^18^ provide evidence that carbonates could be stable and present in the lower mantle. Thus, to fully evaluate carbonate stability in the lower mantle, it is necessary to study carbonates under relevant pressures, temperatures, and oxygen fugacities, as set by lower mantle mineral phase assemblages. Several experimental studies of carbonate reactions under lower mantle conditions exist. Dorfman et al.^20^ reacted (Mg,Ca)CO_3_ and Fe to produce a mixture of diamond, Fe_7_C_3_, and (Mg,Fe)O and found that CaCO_3_ was preserved under deep mantle conditions. Zhu et al.^22^ found that reactions between MgCO_3_ and Fe produce diamond and that the rate of the reaction depends positively on temperature and negatively on pressure. Lv et al.^21^ reacted carbonate and silicate together and found a reversible cation exchange reaction that preserved CaCO_3_ over MgCO_3_. Theoretical studies examining carbonate reactions in lower mantle phase assemblages are scarcer. Pickard & Needs^34^ find that in a reaction with MgSiO_3_, CaCO_3_ becomes more stable than MgCO_3_ above ∼100 GPa. Zhang et al.^35^ find that in the same reaction, MgCO_3_ is preserved throughout the entire range of the lower mantle. They also find that carbonates readily react with SiO_2_ and decarbonate to form silicates and CO_2_. However, more experimental and theoretical petrologic studies of carbonates at lower mantle conditions are necessary, as the behavior of lower mantle minerals is complicated by phase transitions, coordination changes, and melting that could significantly affect their chemical and physical properties.

Here, we present results from experiments on carbonate-metal redox reactions in the laser-heated diamond anvil cell to determine the stable carbon-bearing phase in an example of a lower mantle phase assemblage. We react either magnesite (MgCO_3_) or calcite (CaCO_3_) with Fe_3_Si at pressures up to 123 GPa and temperatures up to 3200 K. Iron-silicon alloy is selected as a reactant due to the presumed presence of Fe in the lower mantle through disproportionation reactions^36^ and as a candidate phase in the Earth’s outer core.^37,38^ By selecting Fe_3_Si as a reactant, we also introduce silicon into the system, allowing us to elucidate the role of silicon in carbonate-metal reactions by comparison to the work of Dorfman et al.^20^ and Zhu et al.^22^

## Methods

The CaCO_3_ used in this study was obtained either from Sigma-Aldrich (Lot D380C128, anhydrous) or from an optically pure natural calcite sample of composition (Ca_0.99992_Sr_0.00008_)CO_3_. Natural samples of magnesite were from Snarum, Norway (University of Chicago mineral collection, number 3733) with the composition (Mg_0.95_Ca_0.03_Fe_0.02_)CO_3_. Carbonate compositions were confirmed by X-ray fluorescence, and structures were confirmed by Raman spectroscopy performed at the University of Chicago. The Fe–Si alloy (Fe_3_Si) used in the study was obtained from Keokuk Electro-Metals Company. It contained 15.9 wt % silicon (Fe_0.73_Si_0.27_ by mole), based on electron microprobe measurements at the University of Maryland, and was chemically homogeneous.^37^ All starting materials were individually ball milled for 1.5 h at 20 Hz in a tungsten carbide (WC) capsule, and grain sizes after ball milling were less than 1 μm.

Samples were then loaded using one of the following 2 configurations: (1) CaCO_3_ and Fe_3_Si were blended together in a 1:1 molar ratio in a WC capsule using a ball mill at 20 Hz for 1.5 h. The mixture was then pressed between diamonds to form platelets 5–10 μm thick and approximately 50 μm in diameter. Argon, used as a quasi-hydrostatic pressure medium and pressure standard, was loaded cryogenically as a liquid; (2) Fe_3_Si was pressed between diamonds to form foils 5–10 μm thick and approximately 50 μm in diameter, which were loaded between powdered samples of either CaCO_3_ or MgCO_3_, with no additional pressure medium. Both configurations used rhenium gaskets preindented to ∼28 GPa in symmetric diamond anvil cells (DACs), and diamonds with culet sizes of 250 or 150 μm were used depending on the pressure range. The prepared sample assemblies were baked for 30 min at 100 °C prior to closing the DACs to mitigate the effect of water absorption.

X-ray diffraction (XRD) experiments were performed at 13-ID-D (GSECARS) at the Advanced Photon Source, Argonne National Laboratory. Samples were compressed to a target pressure, and XRD was used to measure the sample before, during, and after laser heating experiments. We observed no shrinkage of the sample chamber with increasing pressure due to the presence of either the Ar pressure medium or the carbonate phase. Experiments were performed at pressures between 28 and 123 GPa and temperatures up to 3200 K (see Tables 1 and 2 for experimental conditions). Laser-heating experiments were combined with a monochromatic incident X-ray (λ = 0.3344 or 0.2952 Å) of area 2.5 × 3.5 μm at full-width at half-maximum of the focused spot. Fe_3_Si served as a laser coupler, allowing the carbonate phases to be heated through thermal conduction. The flat top laser heated spot size is about 10 μm in diameter,^39^ allowing between 3 and 5 spots to be heated within the same sample, depending on the size of the sample chamber and the geometry of the metal foil. When multiple heating experiments were performed in the same sample, the initial phases present in the new spot were checked using XRD to ensure that there was no overlap between heating spots. Samples were heated from ambient temperature by increasing the laser power and then allowing the sample to sit at the new temperature for approximately 35 s, with an average increase of 115 K for each increase in laser power. Samples were heated beyond the reaction temperature and held at the maximum temperature for a few minutes. Samples were then either cooled slowly by decreasing laser power and allowing the samples to sit for 20 s at each new temperature, with an average decrease of 194 K for each decrease in laser power, or immediately quenched from high-temperature. Heating and cooling conditions for each sample are reported in Tables 1 and 2. Sample-to-detector distances and geometry were calibrated using the NIST standard LaB_6_. The integration of diffraction patterns to produce 2θ plots was performed using DIOPTAS.^40^ Positions of individual diffraction peaks were determined by using PeakFit (Systat Software) by fitting individual peaks to single Gaussian curves. In samples using an argon medium, pressures in laser-heated samples were determined using the Ross et al.^41^ equation of state for argon with temperatures adjusted for axial temperature gradients within the insulator as described by Campbell et al.^42^ Pressure errors were calculated using the mean square distance of the fitted *d*-spacing to the predicted *d*-spacing from the Ross et al.^41^ equation of state. For samples without an argon pressure standard, pressures were measured before and after laser heating using diamond edge Raman,^43^ with additional thermal pressure (6–12% of the preheating pressure) estimated based on previous experiments with a similar geometry.^44^ Pressure errors were estimated from the difference in measured pressure before and after heating, and a temperature error of ±100 K was estimated from the gray body approximation used to measure temperatures during laser heating.

**Table 1:** Experimental Conditions for the CaCO_3_ Reaction^a^

sample	pressure before reaction (GPa)	maximum temperature (K)	heating time (minutes)	cooling time (minutes)	pressure medium	geometry	reactants	observed new phases
AD7	28	2419	14.5	4.5	Ar	1:1 mixture	Fe_3_Si, CaCO_3_-VII, CaCO_3_ (aragonite)	CaSiO_3_, Fe_3_C, FeO, CaO, SiO_2_, Fe_7_C_3_
AD1	32	1632	12.5	1.5	Ar	1:1 mixture	Fe_3_Si, CaCO_3_-VII	CaSiO_3_, Fe_3_C, FeO, CaO, SiO_2_, Fe_7_C_3_
AD7	38	2544	12	3	Ar	1:1 mixture	Fe_3_Si, CaCO_3_ (postaragonite)	CaSiO_3_, Fe_3_C, FeO, CaO, SiO_2_, Fe_7_C_3_
AD1	39	2172	9.5	2	Ar	1:1 mixture	Fe_3_Si, CaCO_3_ (postaragonite)	CaSiO_3_, Fe_3_C, FeO, CaO, SiO_2_, Fe_7_C_3_
AD22	40	1622	12.5	0	None	metal sandwich	Fe_3_Si, CaCO_3_ (postaragonite)	CaSiO_3_, Fe_3_C, FeO, CaO, SiO_2_, Fe_7_C_3_
AD1	47	2423	7.5	2	Ar	1:1 mixture	Fe_3_Si, CaCO_3_ (postaragonite)	CaSiO_3_, Fe_3_C, FeO, CaO, SiO_2_, Fe_7_C_3_
AD7	52	2843	6.5	4.5	Ar	1:1 mixture	Fe_3_Si, CaCO_3_ (postaragonite)	CaSiO_3_, Fe_3_C, FeO, CaO, SiO_2_, Fe_7_C_3_
AD7	54	2753	11	4.5	Ar	1:1 mixture	Fe_3_Si, CaCO_3_ (postaragonite)	CaSiO_3_, Fe_3_C, FeO, CaO, SiO_2_, Fe_7_C_3_
AD1	57	2567	23	3.5	Ar	1:1 mixture	Fe_3_Si, CaCO_3_ (postaragonite)	CaSiO_3_, Fe_3_C, FeO, CaO, SiO_2_, Fe_7_C_3_
AD58	79	2970	23	11	None	metal sandwich	Fe_3_Si, CaCO_3_ (postaragonite)	CaSiO_3_, Fe_3_C, FeO, CaO, SiO_2_, Fe_7_C_3_
AD58	103	3107	17.5	7	None	metal sandwich	Fe_3_Si, CaCO_3_ (*P*2_1_/*c*)	CaSiO_3_, Fe_3_C, FeO, CaO, SiO_2_, Fe_7_C_3_
AD58	123	3208	9.5	0	None	metal sandwich	Fe_3_Si, CaCO_3_ (*P*2_1_/*c*)	CaSiO_3_, Fe_3_C, FeO, CaO, SiO_2_, Fe_7_C_3_

a Cooling times of 0 indicate that the sample was quenched from high-temperature.

**Table 2:** Experimental Conditions for the MgCO_3_ Reaction

sample	pressure before reaction (GPa)	maximum temperature (K)	heating time (minutes)	cooling time (minutes)	pressure medium	geometry	reactants	observed new phases
AD54	33	2214	9	3	none	metal sandwich	Fe_3_Si, MgCO_3_	MgSiO_3_, Fe_3_C, Fe_7_C_3_, MgO, FeO, SiO_2_
AD59	42	2706	32.5	4.5	none	metal sandwich	Fe_3_Si, MgCO_3_	MgSiO_3_, Fe_3_C, Fe_7_C_3_, MgO, FeO, SiO_2_
AD54	46	2571	7.5	2.5	none	metal sandwich	Fe_3_Si, MgCO_3_	MgSiO_3_, Fe_3_C, Fe_7_C_3_, MgO, FeO, SiO_2_
AD54	53	2526	5.5	13	none	metal sandwich	Fe_3_Si, MgCO_3_	MgSiO_3_, Fe_3_C, Fe_7_C_3_, MgO, FeO, SiO_2_
AD59	54	2785	11.5	3.5	none	metal sandwich	Fe_3_Si, MgCO_3_	MgSiO_3_, Fe_3_C, Fe_7_C_3_, MgO, FeO, SiO_2_
AD54	63	2451	4.5	3	none	metal sandwich	Fe_3_Si, MgCO_3_	MgSiO_3_, Fe_3_C, Fe_7_C_3_, MgO, FeO, SiO_2_
AD59	66	2733	19.5	5	none	metal sandwich	Fe_3_Si, MgCO_3_	MgSiO_3_, Fe_3_C, Fe_7_C_3_, MgO, FeO, SiO_2_
AD59	77	3040	11	3.5	none	metal sandwich	Fe_3_Si, MgCO_3_	MgSiO_3_, Fe_3_C, Fe_7_C_3_, MgO, FeO, SiO_2_

For the sample recovery experiment, sample AD22 was decompressed from 40 GPa and secured within the gasket to a 1.3 cm aluminum SEM pin stub. The sample was coated with a thin (∼10 nm) layer of carbon to provide a conductive surface for electron beam imaging. The sample was sectioned along the axis of compression through the center of the laser-heating spot by using a TESCAN LYRA3 focused ion-beam–scanning electron microscope (FIB–SEM) at the University of Chicago. The sample region was fortified by a platinum strip deposited on top of the section, attached to a tungsten needle, and removed from the sample chamber. The FIB section was secured to a copper TEM grid and thinned to less than 0.5 μm. Chemical analysis was performed on the same instrument using an Oxford energy dispersive X-ray spectrometer (EDS) equipped with two XMax 80 mm^2^ silicon drift detectors and Aztec software using a 10 keV electron beam.

## Results

We heated CaCO_3_, or MgCO_3_, and Fe_3_Si to produce the following carbon and silicon exchange reactions
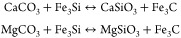
1

Carbon and silicon exchange to form either bridgmanite, or davemaoite, and iron carbide. In this reaction, an oxidized form of carbon, carbonate, is on the left side of the equation, while a reduced form of carbon, carbide, is on the right side of the equation. Evaluating whether this reaction proceeds under the pressure and temperature conditions of the mantle allows one to determine whether carbonate or carbide is the stable carbon-bearing phase in the mantle.

In the following sections, results from XRD experiments and chemical analysis of a recovered sample are presented in separate sections. Although the data are reported in this way for the sake of clarity, our interpretations are based on a holistic assessment of all available data using multiple characterization techniques.

### XRD Results

**Figure 1:**
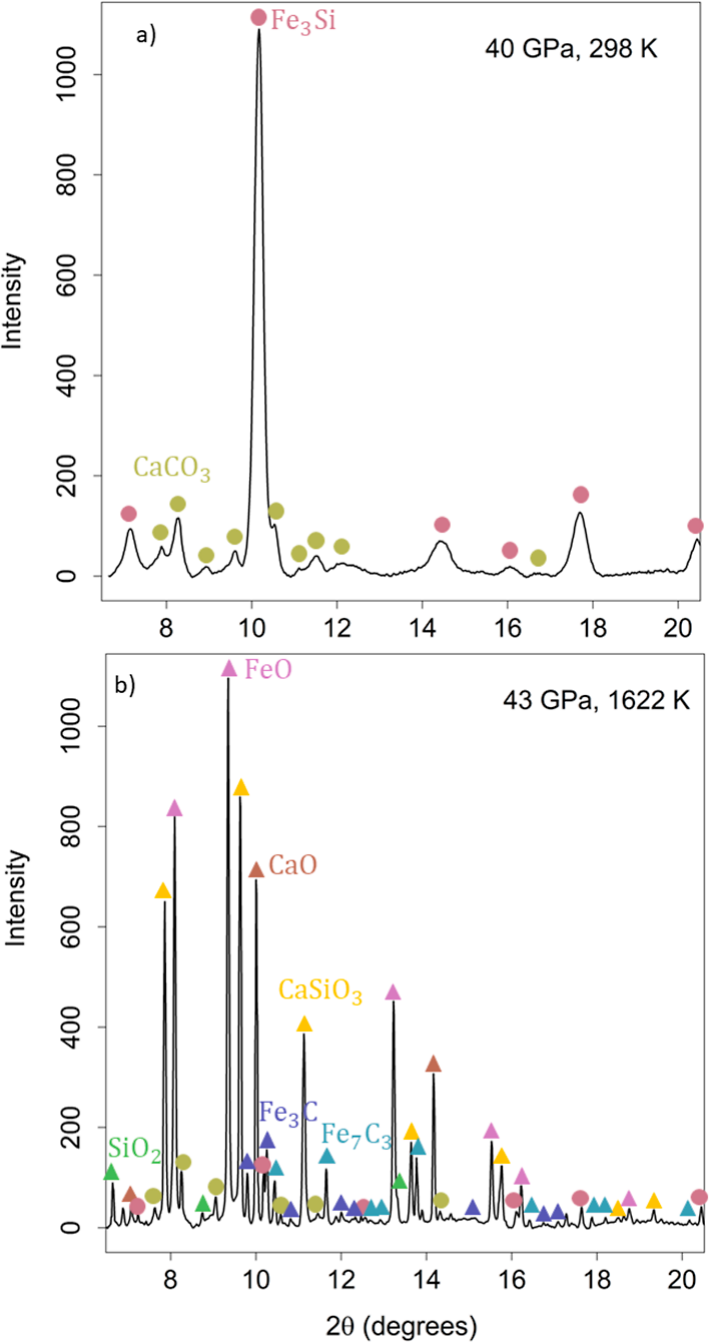
XRD patterns for the reaction CaCO_3_ + Fe_3_Si at (a) 40 GPa and 298 K (before reaction) and (b) 43 GPa and 1622 K (after reaction) for sample AD22. Starting materials are labeled with circles, while new phases formed upon reaction are labeled with triangles. Before the reaction, only the starting materials, Fe_3_Si (B2) and CaCO_3_ (postaragonite), are identified. After the reaction, the new phases CaSiO_3_ (davemaoite) and Fe_3_C are present in addition to the intermediate phases FeO, CaO, SiO_2_ (stv), and Fe_7_C_3,_ and unreacted Fe_3_Si and CaCO_3_. These phases are also identified in the SEM images.

**Figure 2:**
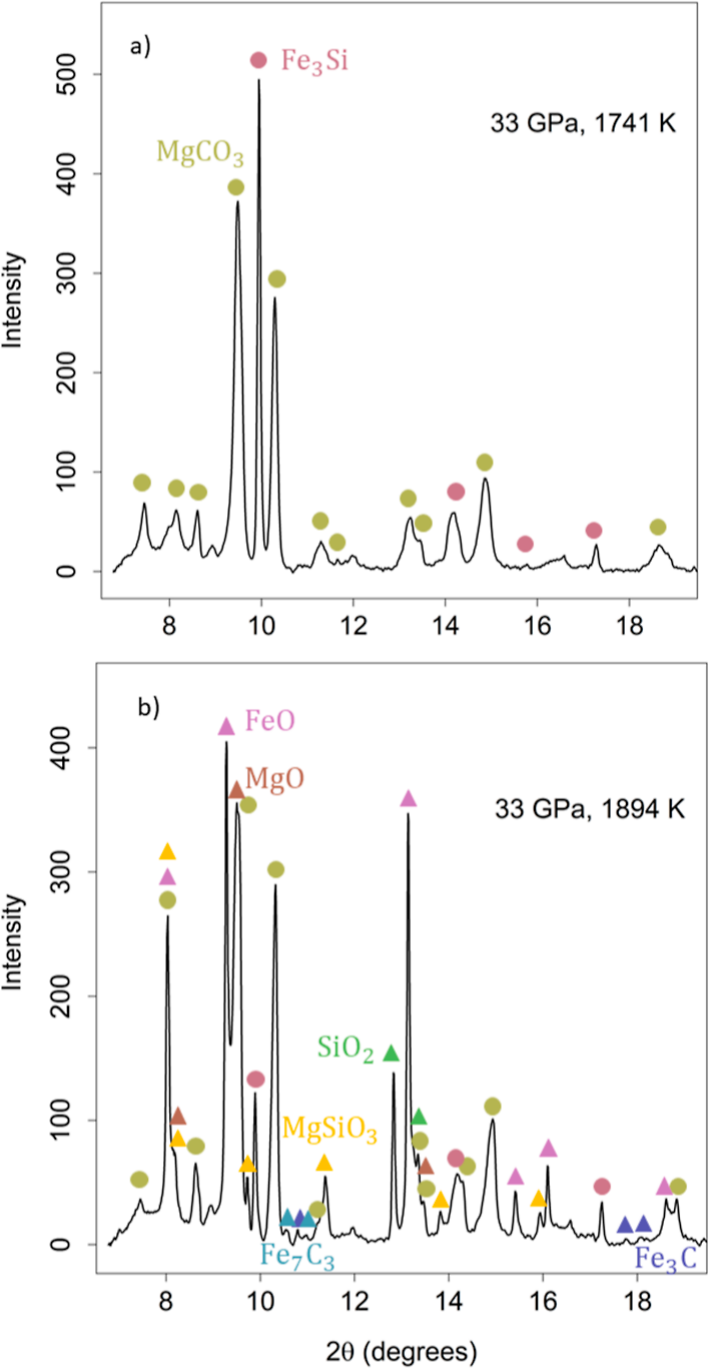
XRD patterns for the reaction MgCO_3_ + Fe_3_Si at (a) 33 GPa and 1741 K (before reaction) and (b) 33 GPa and 1894 K (after reaction) for sample AD54. Starting materials are labeled with circles, while new phases formed upon reaction are labeled with triangles. Before the reaction, only the starting materials Fe_3_Si (B2) and MgCO_3_ are identified. After the reaction, the new phases, MgSiO_3_ (bridgmanite) and Fe_3_C, are present in addition to the intermediate phases FeO, MgO, SiO_2_ (stv), and Fe_7_C_3_ and to the unreacted starting material.

**Figure 3:**
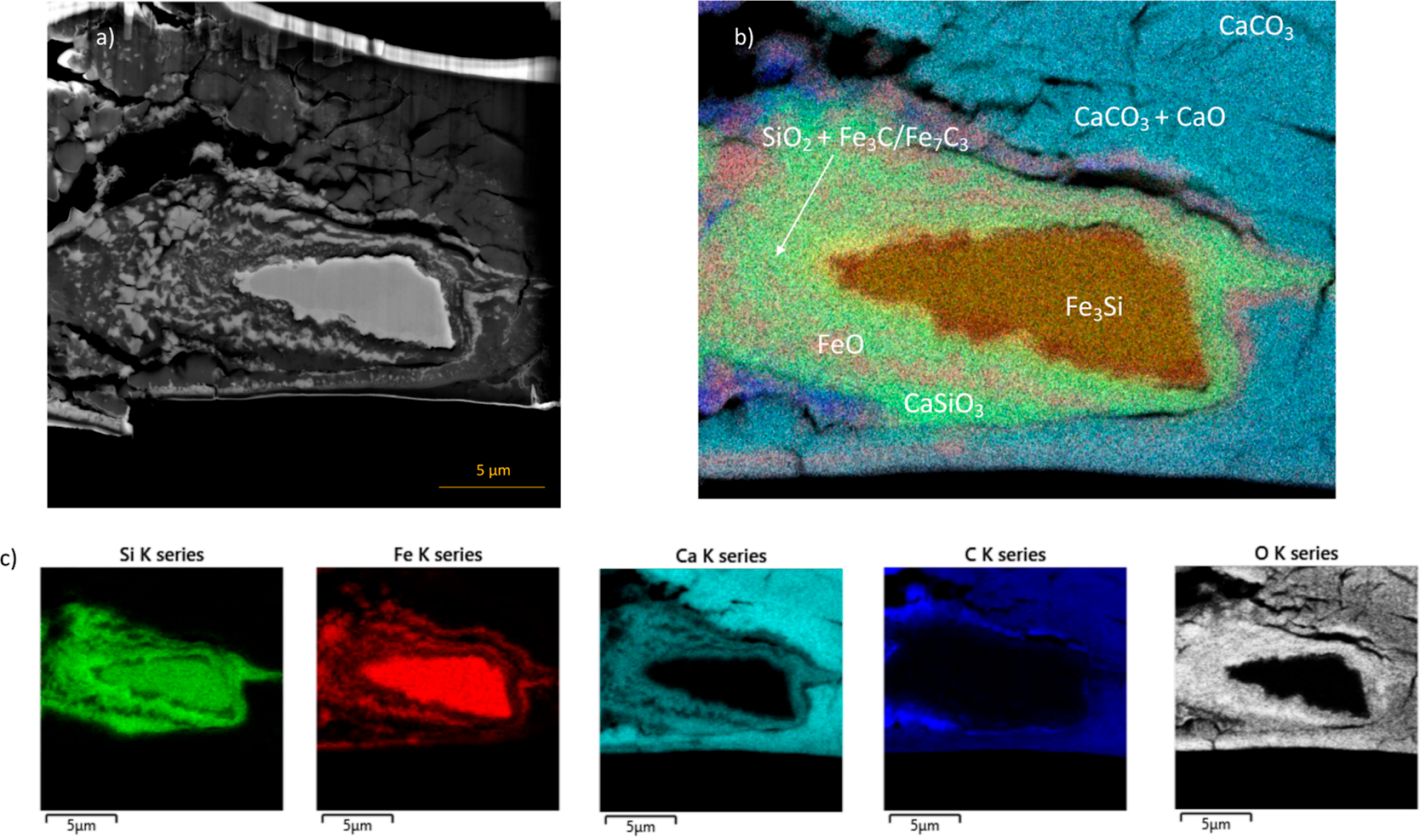
SEM image of the CaCO_3_ + Fe_3_Si reaction, which was quenched from 1613 K at 40 GPa (sample AD22). (a) BSE image of the recovered sample, which is mounted on a TEM grid. (b) EDS map of the recovered sample, with identified compounds labeled. Unreacted Fe_3_Si is surrounded by a fine-grained quench matrix containing CaSiO_3_, FeO, Fe_3_C, Fe_7_C_3,_ and SiO_2_. Surrounding the quench matrix is a halo containing CaCO_3_ and CaO. The reacted material is surrounded by unreacted CaCO_3_. (c) EDS maps of individual elements.

**Figure 4:**
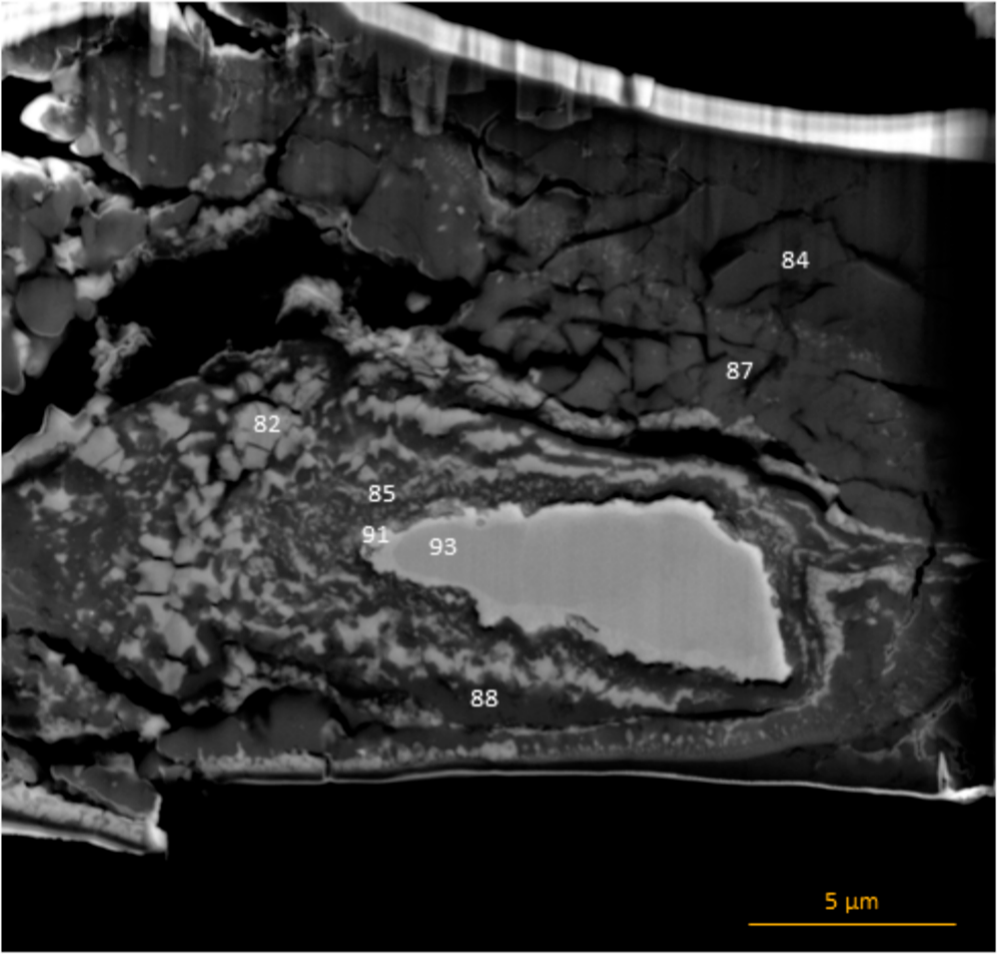
Locations of EDS point analyses for Table 3 (sample AD22).

Both reactants and products were identified through powder XRD (Figures 1 and 2) and EDS measurements (Figures 3 and 4), except for iron carbide phases, which were identified only through XRD due to the difficulty of obtaining quantitative measurements of carbon-bearing phases through EDS. Figure 1 displays example diffraction patterns for the CaCO_3_ reaction at 40 GPa before (Figure 1a) and after (Figure 1b) the reaction (sample AD22). For clarity, only a single phase is assigned to each peak in the pattern, although there is extensive peak overlap resulting from the presence of multiple phases in the system. Before the reaction, peaks from the starting materials, Fe_3_Si and CaCO_3_, are present. Fe_3_Si adopts the B2 structure,^37^ while CaCO_3_ adopts the orthorhombic *P*2_1_2_1_2 postaragonite structure.^5^ After the reaction, many more peaks are present, even though there are also peaks corresponding to unreacted Fe_3_Si and CaCO_3_, indicating that the reaction did not go to completion. The incompleteness of the reaction is evidenced by the SEM image as well (Figure 3), which shows that an axial temperature gradient was present throughout the sample, allowing unreacted material to remain at the cold edges of the sample. However, new peaks corresponding to expected reaction products also appear in Figure 1b, indicating that a reaction occurred even if it did not reach completion. Peaks corresponding to cubic *Pm*3̅*m* CaSiO_3_ (davemaoite)^45^ and orthorhombic *Pnma* Fe_3_C^46^ are present as anticipated. Additionally, peaks corresponding to B1 FeO,^47^ B1 CaO,^48^ SiO_2_ (stishovite),^49^ and orthorhombic Fe_7_C_3_^50^ appear, but only in subsequent XRD patterns at higher temperatures after the initial appearance of CaSiO_3_ and Fe_3_C. The initial formation of CaSiO_3_ and Fe_3_C at the reaction front creates a physical barrier preventing further direct reaction of CaCO_3_ and Fe_3_Si. As the reaction proceeds, CO_2_ and SiO_2_ are formed and diffuse across the reaction barrier to complete the reaction (see EDS Results Section). As a result, FeO, CaO, and Fe_7_C_3_ are intermediate phases that form over the course of the reaction pathway. The oxides FeO and CaO remain spatially separated in the reaction and do not form a single solid solution phase (Figure 1). Based on the observation of these intermediate phases, a plausible reaction pathway is as follows
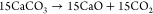
2
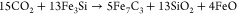
3
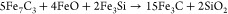
4
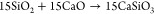
5

Equations 2 through 5 add up to equal 15 of eq 1 and show a complete pathway for the carbon-silicon exchange reaction. First, CaCO_3_ decarbonates to form CaO and CO_2_. CO_2_ diffuses across the reaction barrier, where it reacts with Fe_3_Si to form Fe_7_C_3_, SiO_2_, and FeO. Fe_7_C_3_, FeO, and Fe_3_Si then react to make Fe_3_C and SiO_2_, and SiO_2_ diffuses back across the reaction barrier, where it reacts with CaO to make CaSiO_3_. Because the X-rays probe the entire sample across the axial temperature gradient, the diffraction pattern captures phases at all three stages of the reaction: (1) unreacted starting material (Fe_3_Si and CaCO_3_); (2) intermediate phases that form during the reaction (CaO, Fe_7_C_3_, SiO_2_, and FeO); and (3) completed exchange reaction products (Fe_3_C and CaSiO_3_). Fe_3_C and Fe_7_C_3_ appear as distinct phases in the XRD patterns (Figure S1), and Fe_7_C_3_ is present at all studied pressures. Due to the high proportion of carbon diffusing across the reaction barrier in the form of CO_2_ (eq 3), the reaction with Fe_3_Si results in the initial formation of the more carbon-rich iron carbide phase, Fe_7_C_3_, rather than Fe_3_C as predicted at these pressure conditions by Liu et al.^51^ The experimental conditions and reaction products for all samples of the CaCO_3_ reaction are highlighted in Table 1. CaCO_3_ adopts the CaCO_3_-VII structure^4^ below 38 GPa, the postaragonite structure from 38 to 79 GPa, and the tetrahedrally coordinated *P*2_1_/*c* structure^52^ above 79 GPa. Additional diffraction patterns for the same reaction under different conditions are displayed in Figure S2.

The MgCO_3_ reaction behaves similarly to the CaCO_3_ reaction. Figure 2 displays examples of XRD patterns at 33 GPa before (Figure 2a) and after (Figure 2b) reaction for sample AD54. For clarity, only a few phases are assigned to each peak in the pattern, although there is extensive peak overlap resulting from the presence of multiple phases in the system. In Figure 2a, peaks corresponding to Fe_3_Si and MgCO_3_^53^ are identified. In the diffraction pattern after reaction, peaks corresponding to unreacted starting material are joined by new peaks corresponding to end products MgSiO_3_ (bridgmanite) and Fe_3_C, and intermediate products FeO, MgO, SiO_2_, and Fe_7_C_3_. As in the CaCO_3_ reaction, FeO, MgO, and Fe_7_C_3_ are formed from eqs 2–5, where Ca is replaced by Mg. The experimental conditions and quench products for the MgCO_3_ reaction are identified in Table 2.

### EDS Results

Our XRD data are complemented by the EDS analysis of a recovered sample. A sample of the CaCO_3_ reaction quenched from 1613 K and recovered from 40 GPa (sample AD22) is shown in Figures 3 and 4 (see also Figure S4 and Table S1). Figure 3a is the backscattered electron image, and Figures 3b,c and 4 are EDS maps and point analyses, respectively. Point analysis data are reported in Table 3. Because the sample was carbon-coated and due to the difficulty of measuring carbon through EDS analysis, we do not report quantitative carbon measurements but identify the phases based on the carbon-free measurements and also from the phases present in the XRD results.

**Table 3:** Compositions Obtained From EDS Point Analysis (Sample AD22)^a^

point	O (%)	Si (%)	Ca (%)	Fe (%)	C (%)	phases
82	50.22 (0.40)	2.64 (0.08)	2.66 (0.07)	44.49 (0.30)		FeO + CaSiO_3_
84	52.23 (0.70)	0.31 (0.04)	24.94 (0.14)	0	22.52 (6.98)	CaCO_3_
85	48.97 (0.45)	17.94 (0.13)	4.00 (0.07)	21.45 (0.21)	7.64 (2.37)	SiO_2_ + Fe_3_C/Fe_7_C_3_
87	52.19 (0.70)	0.25 (0.04)	27.42 (0.15)	1.40 (0.07)	18.73 (5.81)	CaCO_3_ + CaO
88	61.64 (0.60)	16.64 (0.11)	18.91 (0.12)	2.80 (0.09)		CaSiO_3_
91	17.58 (0.39)	16.15 (0.19)	0.7 (0.07)	65.57 (0.41)		Fe_3_(Si,O)
93	0	26.26 (0.22)	0	73.74 (0.48)		Fe_3_Si

a All elemental abundances are listed in atomic percent. Standard deviations for the measurements are listed in parentheses. In spots with very low carbon contents, carbon measurements have been excluded due to large errors in quantitative carbon measurements.

From the EDS analyses, we saw a number of different phases across the sample cross-section, revealing the temperature gradient in our samples. The cold edges of the sample contain unreacted CaCO_3_ starting material. Moving closer to the center of the sample, we identified a halo of CaCO_3_, enriched in calcium and iron and depleted in carbon. We interpreted this region as CaO + CaCO_3_, and it marks the reaction front. Carbon is depleted due to the diffusion of CO_2_ across the reaction barrier to react with Fe_3_Si. Carbon is a highly mobile element, as evidenced by the experimentally measured fast diffusion of CO_2_ through carbonates^54^ and the high calculated diffusivities of carbon in silicate melts.^55,56^ We concluded that CO_2_ diffuses quickly through the carbonate material to react with Fe_3_Si at the reaction front, as shown in eq 3. We identified the central metal blob as unreacted Fe_3_Si, which remained in the sample because the reaction did not go to completion. The edges of the metal blob are enriched in Fe and O and depleted in Si (Figure S3b), indicating that Si diffused out of the Fe_3_Si material to form SiO_2_ and then CaSiO_3_. The material surrounding the metal blob formed bands of FeO and CaSiO_3_. The material immediately to the left of the metal blob was directly in the path of the laser and is very fine-grained compared to other regions of the sample due to the higher temperature. Due to the small size of the grains and the resolution limits of the SEM, there is some overlap in the measurement of individual grains. However, from the point analyses, we deduced that the quench matrix consists of a mixture of CaSiO_3_, FeO, SiO_2_, Fe_3_C, and Fe_7_C_3_. Additionally, there are regions of the sample that appear to have unusually high carbon contents, as indicated by the carbon EDS map (Figure 3c). These regions tend to occur around cracks in the sample, and we attribute the high carbon content around the cracks to the deposition of carbon resulting from the platinum deposition process, from environmental carbon contamination, or from carbon diffusion and segregation from the diamond anvil. Combined with the XRD results, the EDS results confirm the carbon and silicon redox exchange reaction (eq 1) as well as the intermediary steps of the reaction (eqs 2–5).

### Reaction Maps

**Figure 5:**
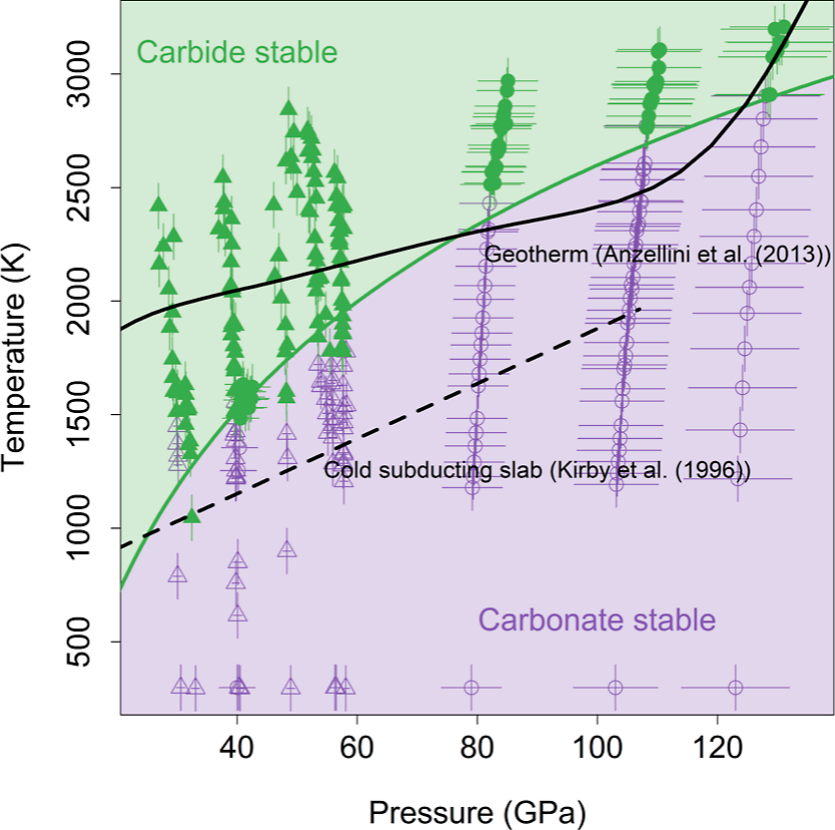
Reaction map illustrating the conditions under which the CaCO_3_ reaction products, CaSiO_3_ and Fe_3_C, are stable (green) and the conditions under which the CaCO_3_ reactants, CaCO_3_ and Fe_3_Si, are stable (purple). Experiments conducted within an argon pressure medium are plotted with triangles, while experiments conducted with a metal sandwich geometry are plotted with circles. Pressures during laser heating are determined from the argon equation of state^41^ or from a thermal pressure estimate based on previous experiments conducted with similar geometry.^44^ The green curve is the fit to the reaction temperature and is compared to the Anzellini et al.^61^ geotherm and the Kirby et al.^62^ subducting slab geotherm.

**Figure 6:**
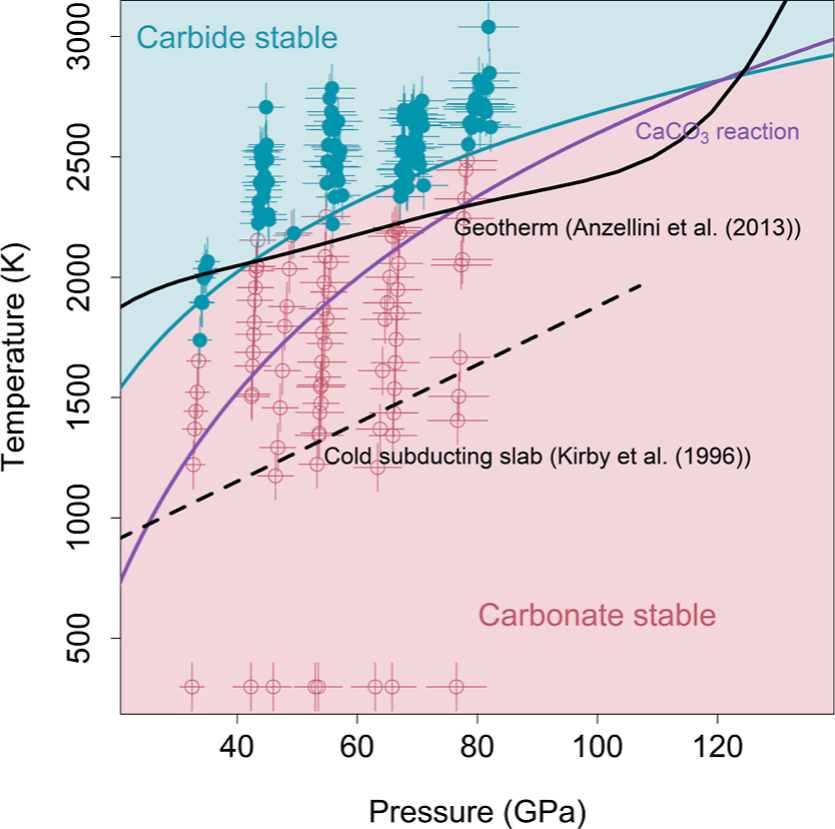
Reaction map illustrating the conditions under which the MgCO_3_ reaction products, MgSiO_3_ and Fe_3_C, are stable (blue) and the conditions under which the MgCO_3_ reactants, MgCO_3_ and Fe_3_Si, are stable (red). Pressures during laser heating are determined from a thermal pressure estimate based on previous experiments conducted with a similar geometry.^44^ The blue curve is the fit to the reaction temperature and is compared to the Anzellini et al.^61^ geotherm, the Kirby et al.^62^ subducting slab geotherm, and the CaCO_3_ reaction (purple line).

Based on the identification of phases in the XRD patterns, we constructed reaction maps for this particular mantle phase assemblage (Figures 5 and 6). The reaction maps illustrate regions in the pressure and temperature space where the products of eqs 1–5 are observed. The boundaries of these regions cannot be identified as phase boundaries, as the experiments were held in a temperature gradient and the products and reactants did not reach equilibrium. Thus, the reaction boundary could be influenced by other factors, such as the heating duration and the initial distribution of products and reactants across the gradient. Nevertheless, the appearance of these phases in our experiments demonstrates that the carbon-silicon exchange reaction progresses at certain pressure and temperature conditions, indicating the stability of the iron carbide + perovskite phase assemblage. Since these reactions were produced upon stepped increases in temperature, the observed reaction temperature must be above the equilibrium phase boundary, indicating that our identified reaction temperatures are an upper bound on the phase boundary.

The perovskite phase always appears among the first new phases formed during the reaction and is the most obvious phase to identify in the diffraction pattern, making it a useful indicator of the occurrence of the exchange reaction. We have calculated the unit cell volumes of both perovskite phases from the XRD patterns, compared them to thermal equations of state^57−59^ (Figures S5 and S6), and found good agreement with previous results. We used the first appearance of perovskites in the pattern to map out reaction temperatures above which the carbonate phase is inferred to no longer be stable. The temperature measured during the diffraction experiments is the temperature at the optically opaque surface. At the moment of reaction, the optically opaque surface is the unreacted Fe_3_Si starting material, and the temperature measured corresponds to the temperature at the center of the sample where the unreacted Fe_3_Si sits (Figure 3). This depth within the sample is also the depth at which we observed the exchange reaction occurring in the SEM image, indicating that the measured temperature corresponds to the temperature of the reaction. In Figure 5, points above the reaction temperature are plotted with filled green symbols, while points below the reaction temperature are plotted with open purple symbols (Tables S2 and S3). Attempts to reverse the reaction through cooling the experiment (lowering the laser power) were unsuccessful, as one would expect based on the reaction sequence preserved in the sample, as illustrated in Figure 3 and discussed above. Thus, only the data points collected upon heating the sample are plotted in Figures 5 and 6 to best showcase the exchange reactions. Experiments in argon have minimal thermal pressure, except at high-temperature when the argon pressure medium begins to melt.^60^ Experiments conducted with the metal sandwich geometry have larger thermal pressures, estimated to be between 6 and 12% of the preheating pressure at the highest temperatures based on previously conducted experiments with similar geometry.^44^ We fit the observed reaction temperatures to a logarithmic function (green line) of the form *T* = *a*+*b* × ln(*P*), which allows us to map out the regions where carbonate is observed to be the stable carbon-bearing phase (purple) and where carbide is observed to form (green). The fit parameters for this reaction are *a* = −2824 ± 461 K and *b* = 1177 ± 115 K/GPa. The temperature at which the reaction was observed increases with pressure, indicating that in this particular phase assemblage, the carbonate phase is stabilized to higher temperatures with increasing depth in the mantle. We compared our reaction line to an example average mantle geotherm^61^ and a cold subducting slab geotherm,^62^ which allowed us to contextualize our results with various pressure and temperature conditions that may be present in the Earth’s interior. Comparing our reaction line to an average mantle geotherm,^61^ we found that the lines cross initially at ∼76 GPa and again at ∼124 GPa. At shallow- to midmantle depths, the geotherm temperature is greater than the reaction temperature, leading to the formation of carbide phases. At midmantle to lowermost mantle depths, the geotherm temperature is colder than the reaction temperature, and it is possible that carbonate is the stable carbon-bearing phase, depending on the location of the phase boundary relative to our observed reaction temperatures. At lowermost mantle depths, the mantle temperature may be hotter than the reaction temperature, and although there is uncertainty about the geotherm and the extrapolated reaction temperature, it is possible that carbide phases are once again stable near the core-mantle boundary. Additionally, as carbonates are key components of subducting slabs, we examine carbonate stability along a subducting slab geotherm.^62^ We find that in a cold subducting slab, CaCO_3_ may become the stable carbon-bearing phase just past the transition zone and remain the stable phase throughout the lower mantle (Figure 5). Although there is uncertainty in the phase relations due to the fact that our experiments did not reach equilibrium, our findings are indicative of increased carbonate phase stability at greater depths in the Earth’s mantle than previously suspected, even in the presence of metal.

The data for the MgCO_3_ reaction is plotted in Figure 6 and is similar to the CaCO_3_ reaction data. We identified the reaction temperature by the appearance of bridgmanite in the diffraction pattern. Points above the reaction temperature are plotted with filled blue circles, and points below the reaction temperature are plotted with open red circles. We fit the reaction temperature to a logarithmic function (blue line) and have shaded the regions where carbide is inferred to be stable in blue and the regions where carbonate is presumed to be stable in red. The fit parameters for this reaction are *a* = −647 ± 402 K and *b* = 723 ± 98 K/GPa. The temperature at which the reaction is observed to occur increases with pressure, indicating that the MgCO_3_ phase is stabilized to increasingly high-temperatures with depth in the mantle. Comparing this reaction line to the Anzellini et al.^61^ geotherm, we found two crossover points at ∼41 and ∼124 GPa. In this system, iron carbide is the stable carbon-bearing phase in the shallow and lowermost lower mantle, while MgCO_3_ may be the stable carbon-bearing phase through much of the lower mantle, depending on how much our observed reaction temperatures overshoot the phase boundary. Along a cold subducting slab geotherm,^54^ temperatures are sufficiently cold to prevent the exchange reaction from occurring, and our experiments suggest that MgCO_3_ is stable in the subducting slab throughout the lower mantle. Additionally, we compared the MgCO_3_ reaction directly to the CaCO_3_ reaction (the purple line in Figure 6). The MgCO_3_ line first crosses the average mantle geotherm around 25 GPa lower than the first crossing for the CaCO_3_ reaction, indicating that MgCO_3_ is stabilized at a shallower depth than CaCO_3_. Additionally, the MgCO_3_ line lies above the CaCO_3_ line until the two lines cross at ∼121 GPa. Thus, we expect CaCO_3_ to replace MgCO_3_ as the stable carbonate phase in the lowermost mantle. Since our MgCO_3_ reaction data must be extrapolated above ∼80 GPa and there is uncertainty in the phase boundary for these systems, the exact pressure at which this crossover occurs is ambiguous. However, the crossover of the two lines is suggestive of switching stability regimes between the two carbonates and is in agreement with recent work.^20,21,34^

## Discussion

The progression of the carbon–silicon exchange reaction at various pressures and temperatures indicates that in this particular phase assemblage carbonate may be the stable carbon-bearing phase for much of the lower mantle even at average lower mantle temperatures, relative to the carbide + silicate assemblage. Along a cold subducting slab geotherm,^62^ we find that carbonates may be preserved within the subducting slab throughout the entire lower mantle. Previous studies of carbonates at the pressure and temperature conditions of the lower mantle indicate that carbonates primarily melt,^1,8^ decarbonate,^9,11^ or are reduced to diamond or carbide via redox freezing.^14,63^ However, many processes dictate the stability of carbonates, including reactions with surrounding phases. In this particular phase assemblage, the carbonate phase is favored over more reduced forms of carbon under lower mantle conditions, even in the presumed presence of metal. If such a phase assemblage exists in subducting slabs, this process could be an important mechanism for the transport of carbon into the Earth’s deep interior.

Other lower mantle petrologic studies of carbonates have seen both similar and different results. In both Dorfman et al.^20^ and Zhu et al.,^22^ carbonates reacted with iron to make iron carbides and diamonds. Similarly, in ab initio molecular dynamics studies of a carbonate-silicate-metal melt by Davis et al.,^56,64^ carbonate, silicate, and metallic melts interact to form carbon and iron clusters indicative of dense Fe–C liquids and diamond nucleation. However, in this work, we see no evidence of diamond formation in either the XRD data or the EDS measurements. While we cannot rule out the possibility that small grains of diamond are undetectable by XRD or SEM imaging formed during the course of our reaction, we consider it unlikely that diamond formed in these experiments. Previous experiments by Dorfman et al.^20^ clearly identified nanometer-sized diamonds in their EDS maps. We see no evidence of individual carbon grains in our own SEM images at the same scale. In Lv et al.,^21^ carbonate and silicate undergo a cation exchange reaction, and there is no evidence of carbon reduction in any form. Theoretical studies of the same reaction^34,35^ agree that no diamond formation occurs. It appears that carbonates reduce to diamond under moderately reducing conditions. In this study, the oxygen fugacity of the system is unknown, as it cannot be controlled a priori in diamond anvil cell experiments and cannot be calculated without the equilibrium phase assemblage. However, here, we react carbonates with an iron-silicon alloy, which is more reducing than pure iron metal, allowing our results to be compared to the less-reducing systems of previous experiments. The reaction of carbonates with a stronger reducing agent drives the system toward iron carbide formation rather than diamond formation. Thus, the behavior of carbon is greatly influenced by the presence of other species, and based on the surrounding phase assemblage and redox conditions of the mantle, we might expect a variety of carbon-bearing phases to be stable.

The slope of the carbonate reaction line indicates that carbon becomes less siderophilic with increasing pressure with respect to silicon. Previous low pressure and temperature measurements (less than 20 GPa and 2600 K) of the carbon partition coefficient,^65−68^*D*_C_^metal/silicate^, suggest partition coefficients on the order of 10^2^ and 10^3^ between metal and silicate melts. Partition coefficients of this magnitude indicate that carbon is highly siderophile and imply that the majority of Earth’s carbon was sequestered into the core upon formation. However, measurements conducted at higher pressures and temperatures corresponding to a deep magma ocean^69^ (up to 59 GPa and 5200 K) report *D*_C_ values on the order of 1–100, implying that less carbon may have been sequestered into the core upon formation than previously thought. The decrease in *D*_C_ values by several orders of magnitude with increasing pressure supports the trend observed in this study and implies that more carbon may be present in the lower mantle and at the core-mantle boundary than previously imagined. Additionally, *D*_C_ values are dependent on bulk carbon content and have been shown to increase with increasing carbon concentration.^68^ In the work of Grewal et al.,^70^ the authors report that previous measurements may have overestimated the amount of carbon in the core due to high *D*_C_ values from oversaturation of carbon in the measured systems, further increasing the notion that more carbon may be present in the lower mantle than previously thought.

**Figure 7:**
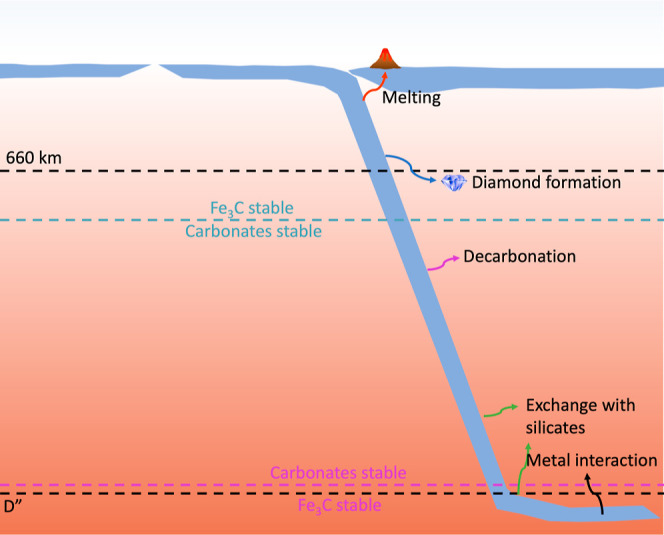
Schematic of possible carbonate fates in the ambient mantle. The dotted blue line denotes the depth at which the stable carbon-bearing phase switches from Fe_3_C to carbonate phases in our example phase assemblage. The purple line denotes the depth at which the stable carbon-bearing phase switches from carbonates back to Fe_3_C. The arrows indicate other processes that carbonates undergo, including melting, redox freezing to form diamond, decarbonation, exchange with silicates, and interactions with metals. Carbonates in a subducting slab would be stable over an even wider depth range within the Earth’s interior.

Figure 7 summarizes some different pathways that carbonates could undergo in the mantle. Previous pathways that have been studied are denoted with arrows on the right side of the figure and include melting to form carbonatitic compositions, diamond formation through redox freezing, decarbonation with free silica, exchange with lower mantle silicates, and interactions with free metal in the mantle or from the core. On the left side of the figure, we denote the regions where Fe_3_C and carbonate phases are presumed to be stable based on the reactions studied in this work. We find that carbonates may be stable in the lower mantle at depths much greater than previously assumed. The stability of the carbonate phases in these exchange reactions indicates the importance of the petrologic context in understanding the behavior of carbon-bearing systems. This work and other recent studies^20−22,34,35^ represent progress in multiphase mantle petrology in the lower mantle, but more studies are necessary to elucidate the stability and reactivity of carbonates in the lower mantle and to fully understand the deep Earth carbon cycle.

## Conclusions

Carbonates in the lower mantle may be produced by a reaction between silicates and iron carbide. Despite the challenges imposed by the thermal gradients in the laser-heated diamond anvil cell, careful comparison of in situ XRD results with SEM examination of the recovered sample can reveal the direction of the exchange reactions studied here. The temperature at which these reactions occur increases with pressure, indicating that the stability field of carbonates also increases with pressure. Additionally, the increased stability of the carbonate phase with depth implies that carbon becomes less siderophilic with increasing pressure with respect to silicon. Comparing the exchange reaction temperatures to a mantle geotherm, we find an initial crossover point where carbonates may become the stable carbon-bearing phase in the mantle over iron carbide. The crossover point occurs at ∼41 GPa in the MgCO_3_ reaction and ∼76 GPa in the CaCO_3_ reaction, and the carbonate phases may remain the stable carbon-bearing phases in the mantle until a second crossover point is reached at ∼124 GPa for both reactions. In this mantle phase assemblage, the carbonate phase may be stabilized throughout much of the lowermost mantle. Along a subducting slab geotherm, we find that carbonates could be the stable carbon-bearing phase throughout the entire lower mantle. Thus, in similar phase assemblages in subducting slabs, carbonates could survive into the lower mantle, where they could participate in reactions with other lower mantle phases.

## Supporting Information Available

Additional diffraction patterns, EDS linescan image and chemical analysis, EDS point analyses, calculated unit cell volumes for perovskite phases, table of thermodynamic data for CaCO_3_ reaction, and table of thermodynamic data for MgCO_3_ reaction (PDF)
