# Supplementary material for: Carbonate-Metal Reactions in the Lower Mantle
Source: ACS Earth Space Chem. 2024 Mar 25;8(4):654–64. doi: 10.1021/acsearthspacechem.3c00101 (PMC11033939; doi:10.1021/acsearthspacechem.3c00101)
Supplement: Supplementary file 1 — sp3c00101_si_001.pdf [file sp3c00101_si_001.pdf]

Supporting Information for

Carbonate-metal reactions in the lower mantle

A.H. Davis[1,*], B.A. Chidester[2], E. Greenberg[3,**], V.B. Prakapenka[3], and A.J. Campbell[1]

[1]Department of the Geophysical Sciences, The University of Chicago, 5734 S. Ellis Avenue, Chicago, IL, 60637, USA
[2]Los Alamos National Laboratory, Los Alamos, NM, 87545, USA
[3]Center for Advanced Radiation Sources, The University of Chicago, 5734 S. Ellis Avenue, Chicago, IL, 60637, USA
[*]Now at the Centre for Planetary Habitability (PHAB), University of Oslo, ZEB building, Sem Sæland vei 2A, Nedre Blindern, 0371, Oslo, Norway
[**]Now at Applied Physics Division, Soreq NRC, Yavne, 81800, Israel

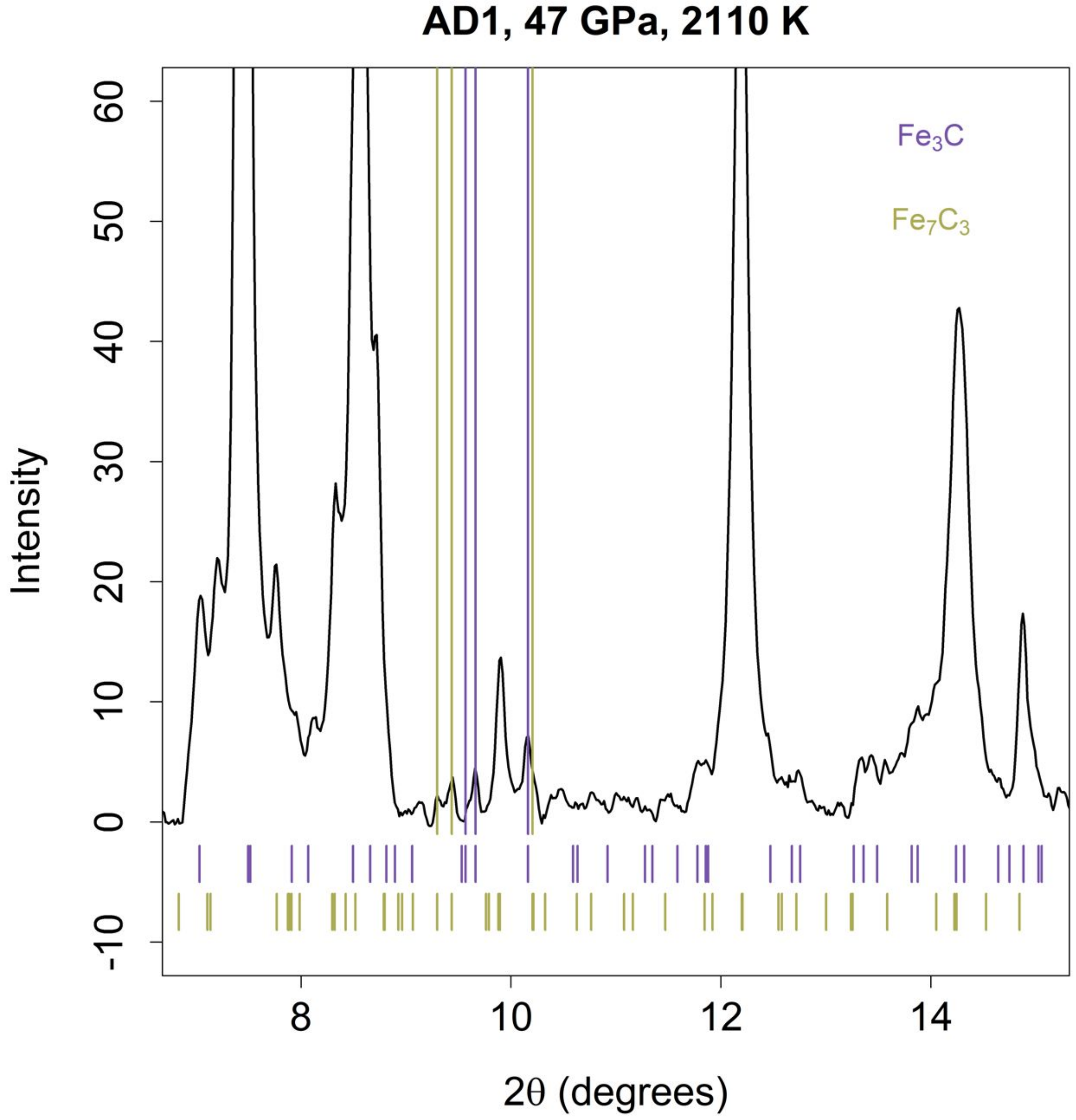

Figure S1. X-ray diffraction pattern for sample AD1 at 47 GPa and 2110 K after the exchange reaction has occurred. Peaks corresponding to the identified Fe_3C and Fe_7C_3 phases are highlighted with purple and green lines, respectively. The (131), (221), and (122) Fe_3C peaks and the (602), (024), and (025) Fe_7C_3 peaks show no overlap with other phases and indicate that both phases are present in the sample. These peaks are highlighted with the large lines spanning the length of the plot.

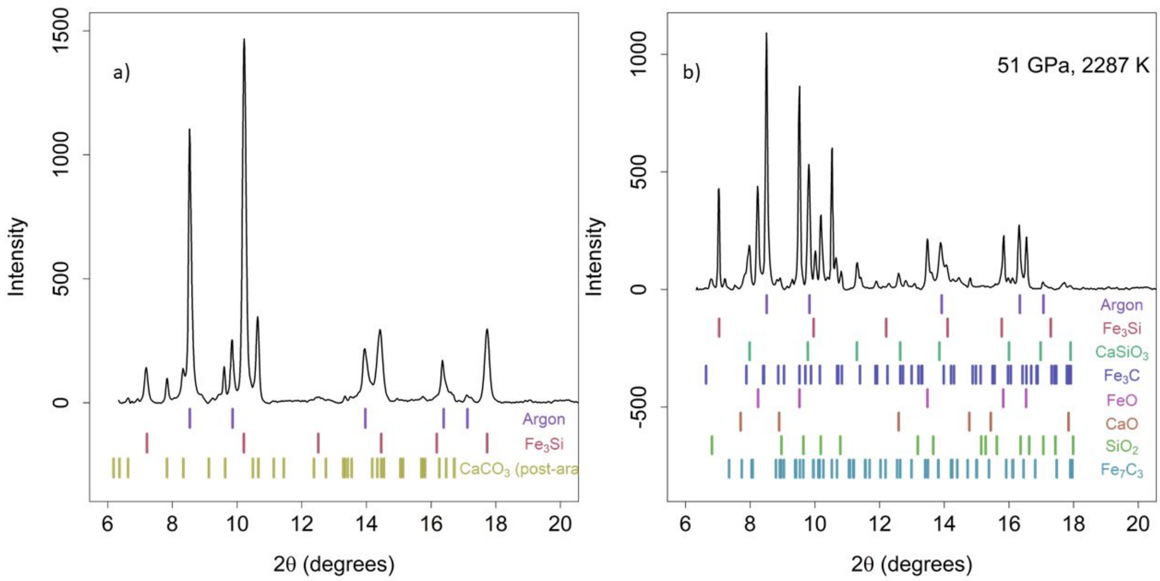

Figure S2. X-ray diffraction patterns for sample AD7 at a) 54 GPa and 1626 K, before a reaction has occurred and at b) 51 GPa and 2287 K, the highest temperature from which the reaction was quenched. Before reaction, only the starting materials, Fe_3Si (B2) and $CaCO_3$ (post-aragonite), and the pressure medium, Ar, are identified. After reaction, the new phases $CaSiO_3$ (perovskite) and Fe_3C are present in addition to the intermediate phases FeO, CaO, SiO_2 (stv), and Fe_7C_3 and to unreacted starting materials and Ar.

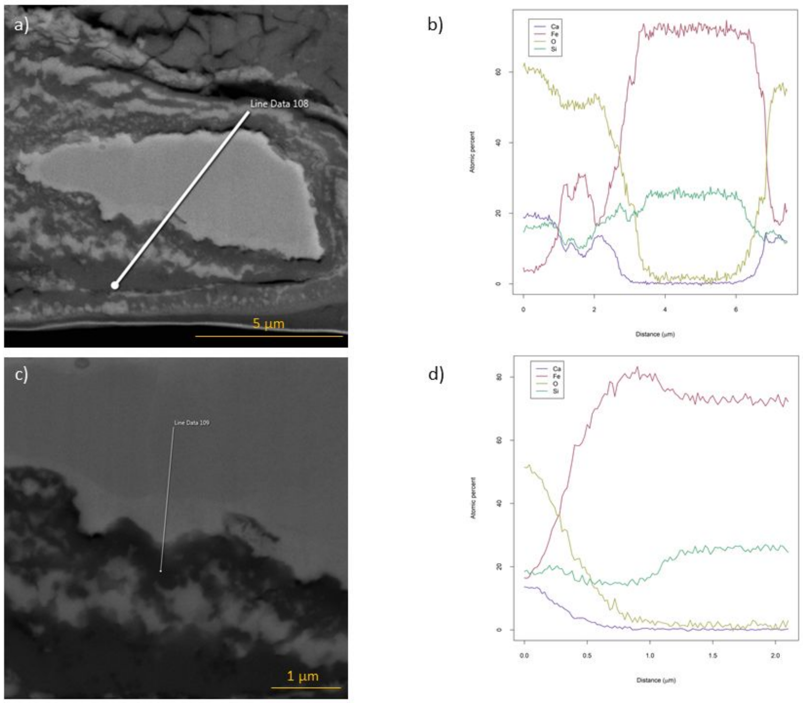

Figure S3. EDS line scans across a-b) the center of the sample and c-d) the edge of the metal blob (sample AD22). The metal blob is unreacted Fe_3Si, and the edge of the metal blob is depleted in silicon.

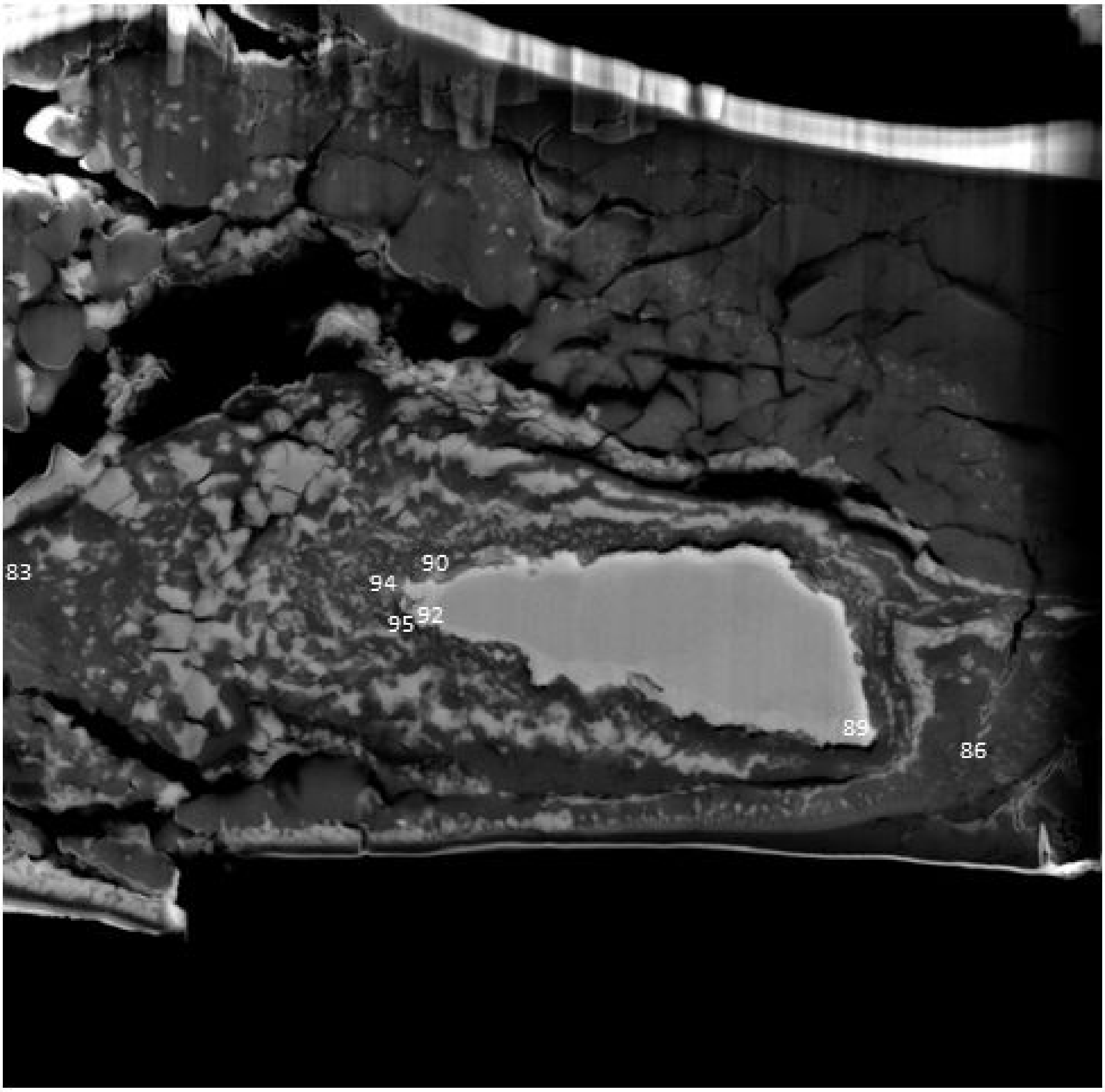

Figure S4. EDS point analyses for Table S1 not included in main text (sample AD22).

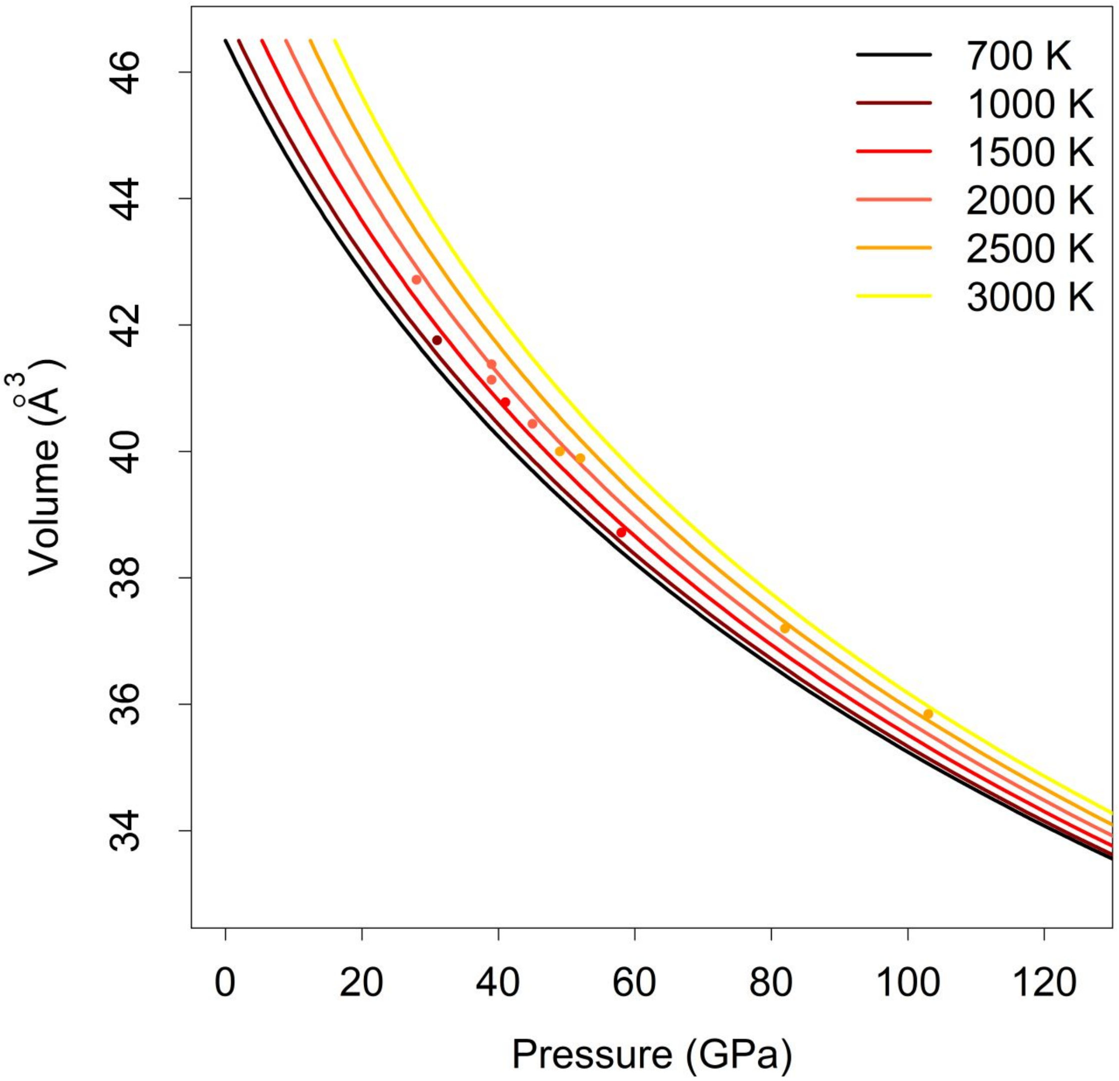

Figure S5. Calculated unit cell volumes for davemaoite as compared to the Noguchi et al.[56] thermal equation of state. Our data (points) are binned by temperature in 500 K increments. Our calculated unit cell volumes are in good agreement with previously calculated results. Deviations to lower volumes are explained by a small amount of solid solution with Fe in the davemaoite structure, as evidenced by the SEM results in Table 3 and Table S1.

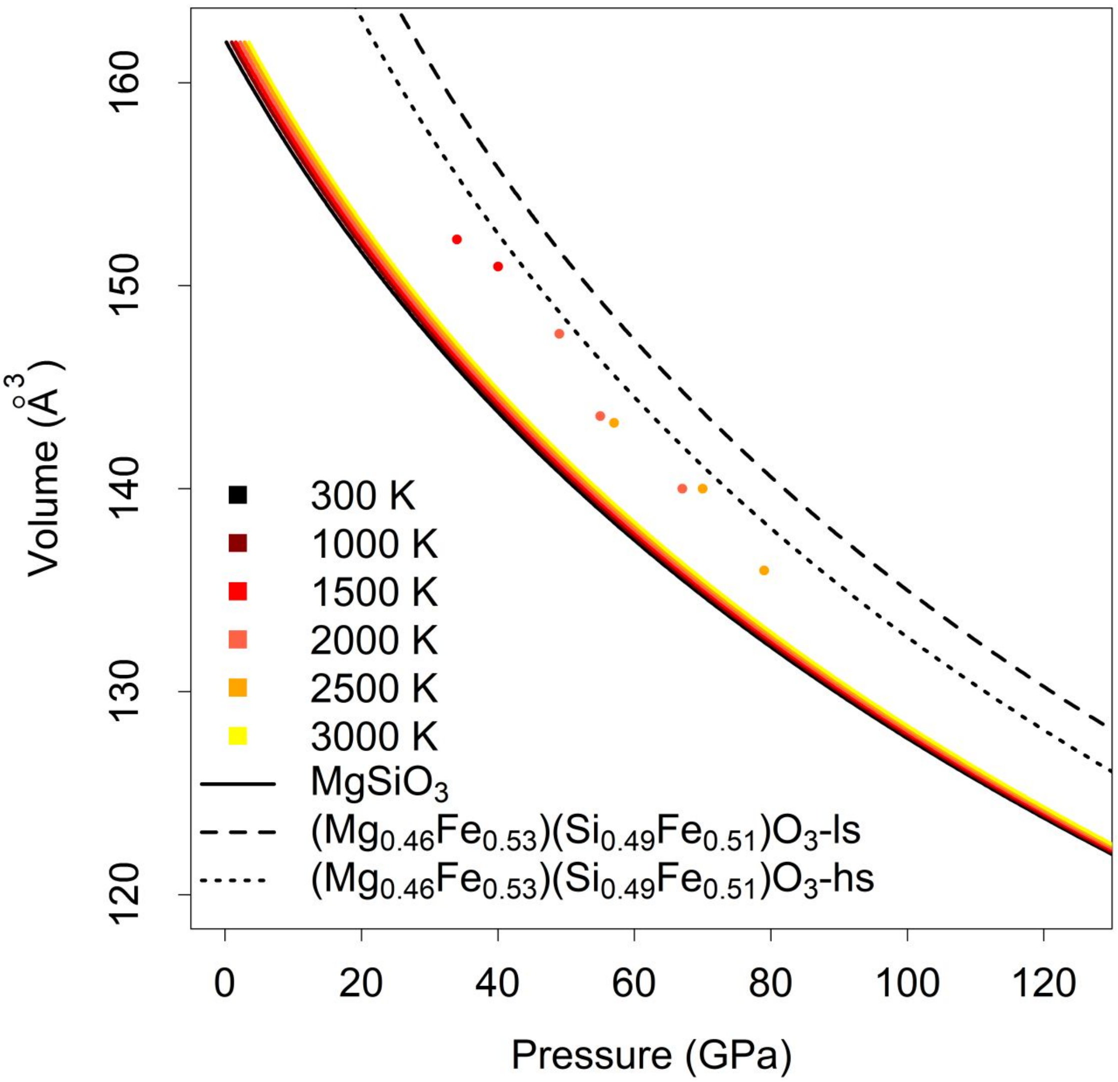

Figure S6. Calculated unit cell volumes for bridgmanite as compared to the pure $MgSiO_3$[57], $(Mg_{0.46}Fe^{3+}_{0.53})(Si_{0.49}Fe^{3+}_{0.51})O_3$ – low spin[58], and $(Mg_{0.46}Fe^{3+}_{0.53})(Si_{0.49}Fe^{3+}_{0.51})O_3$ – high spin[58] equations of state. Our data (points) are binned by temperature in 500 K increments. Our calculated unit cell volumes lie in between the values for pure $MgSiO_3$ and $(Mg_{0.46}Fe^{3+}_{0.53})(Si_{0.49}Fe^{3+}_{0.51})O_3$, indicating Fe substitution into the bridgmanite structure.

Table S1. EDS point analyses for points shown in Figure S2 (sample AD22). Element abundances are reported in atomic percent, and standard deviations are shown in parentheses. Carbon abundances are not included for spots with low carbon abundances, due to high uncertainties in carbon measurements.

Point	*O (%)*	*Si (%)*	*Ca (%)*	*Fe (%)*	*C (%)*	*Phases*
83	58.15 (0.57)	18.78 (0.12)	17.56 (0.11)	5.51 (0.11)		$CaSiO_3$
86	50.35 (0.68)	0.79 (0.04)	23.54 (0.15)	2.36 (0.09)	22.85 (7.08)	$CaCO_3$ + CaO
89	7.79 (0.32)	14.77 (0.18)	1.91 (0.08)	75.54 (0.48)		Fe3(Si,O)
90	40.27 (0.41)	16.94 (0.14)	1.59 (0.07)	31.65 (0.27)	9.55 (2.96)	SiO_2 + Fe_3C/Fe_7C_3
92	10.18 (0.37)	16.59 (0.21)	0.93 (0.07)	72.30 (0.46)		$Fe_3(Si,O)$
94	37.84 (0.40)	17.93 (0.16)	3.09 (0.08)	30.28 (0.27)	10.86 (3.37)	SiO_2 + Fe_3C/Fe_7C_3
95	45.56 (0.46)	19.59 (0.15)	8.16 (0.09)	18.63 (0.06)	8.06 (2.50)	SiO_2 + Fe_3C/Fe_7C_3

Table S2. $CaCO_3$ reaction conditions.

Pressure (GPa)	Reaction Temperature (K)
30	1514
32	1047
40	1508
40	1705
41	1485
48	1574
54	1842
55	1779
58	1778
82	2514
108	2763
128	2908

Table S3. $MgCO_3$ reaction conditions.

Pressure (GPa)	Reaction Temperature (K)
34	1739
44	2224
49	2183
55	2378
56	2390
67	2221
67	2335
78	2552
